# Annotations

**Published:** 1899-05-13

**Authors:** 


### ANNOTATIONS.

### Inspection of Food a Remunerative Undertaking.

One so often finds that sanitary authorities for one reason or another—reasons often connected with supposed economy—are chary about putting in force the powers at their disposal for the suppression of adulteration, that it is well to bear in mind how great is the expense to the community of letting things take their course. Inspection may be expensive, but the daily purchase of adulterated food costs far more in the end. Dr. J. R. Kaye, medical officer to the West Riding, speaking at a meeting of the Yorkshire branch of the Sanitary Inspectors' Association, said that a simple calculation showed that the inhabitants of the West Riding must spend not less than £2,500 a day on milk, equal to the enormous sum of £912,500 per annum. Looking back to the year 1893 they found that of the money thus spent 6·5 per cent. was paid for milk that was adulterated, and, taking the average rate of adulteration at 10 per cent. of added water, it followed that during the year the people paid no less than £5,930 for water which was sold as milk. In 1897, however, adulteration had diminished, but still even for that year the amount paid in the same way for water was £2,281; an astonishing sum, and one which would pay for a great deal of inspection and for a vast number of analyses. Much the same thing has to be said in regard to butter, the substitution of margarine or its admixture with butter often amounting to downright robbery. Coffee again was much adulterated, the chief form of adulteration, namely, the addition of chicory, being clearly fraudulent. All this points to the moral that as a mere commercial undertaking the constant inspection and analysis of food products is the best of all investments for a community, yielding as it does a manifold return on the outlay.

### Tuberculosis and Trains.

Our contemporary, the *Railway Surgeon*, in a recent number, calls attention to the danger of infection by the tubercle bacillus to which railway travellers are exposed. It may be remembered that in 1890 and 1891, when Koch's tuberculin discovery brought large numbers of consumptives to Berlin, it was found that the carriages in which these sufferers had travelled had become infected. Since that time Petri has made elaborate researches to determine whether the tubercle bacillus is commonly present in the dust of railway cars, in what class of carriage it is most frequent, and in what parts of the car the dust has the highest infective properties. As might have been anticipated, the first and second classes were freer than the third and fourth, but dust from all of these was found to contain the specific germ of consumption. Of 91 guinea pigs inoculated with scrapings from the floor 26 were infected. It is obvious that the floors of our railway carriages, covered as they frequently are with expectoration, seldom washed, never freely exposed to air or sunlight, are most fertile breeding grounds for the germs of consumption. The Prussian Minister of Public Works has now made an order that all railway cars shall be cleansed periodically as specified depôts. Carpets and upholstery must be treated with steam, and all wood work washed with potash soap, rinsed with water, and rubbed dry. We would suggest that a further improvement in the hygiene of cars might be brought about by the adoption of leather or other impervious coverings for cushions, by the substitution of linoleum for the present floor carpeting, and by the provision of spittoons for use, especially in the smoking carriages. The chief district sanitary inspector of Perthshire points out that the new Public Health Act for Scotland provides that the local sanitary authority may "make bye-laws for securing the cleanliness and sanitary condition of public conveyances plying within its district," and indicates his intention of framing a code of bye-laws to secure the much-needed improvement. In view of the danger that undoubtedly exists it is desirable that these powers should be extended to all parts of the kingdom, and the travelling public protected from a peril which, although unseen, is as real as that from collision or other railway accident.

### International Council of Women.

The King of France who, in the presence of any evil of undiscovered origin, was accustomed to say "*Cherchez la femme*," would be greatly puzzled if he could revisit this sublunary sphere, and could observe the modern development of feminine activity in the way of overcoming evil with good. The efforts of women in this direction have now extended far beyond the narrow limits of the home; and, in ever-widening circles, are striving to reach those of the habitable globe. We learn from the published versions of a speech by the Countess of Aberdeen, the president of a "Congress of Women," to be held next month in London, that this gathering will have for its objects to receive reports from all countries of associations of women working for the common welfare of the community, and to listen to the counsels of women from almost all parts of the world on subjects with which they are specially qualified to deal. The congress is also intended to "deepen the sense of responsibility in women, and to promote a true sisterhood and a high patriotism among the women of various countries." We will not suggest that there might be occasional friction between the "high patriotism" and the "international sisterhood," but we confess to some curiosity as to the methods by which the objects of the congress are to be attained. The only agency so far revealed is that of talk, which is to be carried on in five sections simultaneously, and is to be continued for ten days. The speakers are to be "chosen by the sectional committee of experts appointed for that purpose"; an arrangement which may be excellent, but which seems to make inadequate provision for the encouragement either of spontaneous eloquence or of the eloquence of hitherto untried orators. We can deeply sympathise with the many ladies who will be likely to re-echo in their minds the sentiment of Juvenal, "*Semper ego auditor tantum? nunquamne reponam,*" and we do not entirely envy the presidents of the several sections, whose painful duty it will be to repress the unauthorised many, and to secure an uninterrupted hearing for the chosen few. Men were present at the preliminary meeting which Lady Aberdeen addressed; but we have not heard whether they will be encouraged, or even tolerated, at the congress itself. A permission to a man to speak would surely be the signal for revolt and open war.

